# *trans*-Complementation of an NS2 Defect in a Late Step in Hepatitis C Virus (HCV) Particle Assembly and Maturation

**DOI:** 10.1371/journal.ppat.1000403

**Published:** 2009-05-01

**Authors:** MinKyung Yi, Yinghong Ma, Jeremy Yates, Stanley M. Lemon

**Affiliations:** 1 Department of Microbiology and Immunology, University of Texas Medical Branch at Galveston, Galveston, Texas, United States of America; 2 Center for Hepatitis Research, Institute for Human Infections and Immunity, University of Texas Medical Branch at Galveston, Galveston, Texas, United States of America; Stanford University School of Medicine, United States of America

## Abstract

Recent studies using cell culture infection systems that recapitulate the entire life cycle of hepatitis C virus (HCV) indicate that several nonstructural viral proteins, including NS2, NS3, and NS5A, are involved in the process of viral assembly and release. Other recent work suggests that Ser-168 of NS2 is a target of CK2 kinase–mediated phosphorylation, and that this controls the stability of the genotype 1a NS2 protein. Here, we show that Ser-168 is a critical determinant in the production of infectious virus particles. Substitution of Ser-168 with Ala (or Gly) ablated production of infectious virus by cells transfected with a chimeric viral RNA (HJ3-5) containing core-NS2 sequences from the genotype 1a H77 virus within the background of genotype 2a JFH1 virus. An S168A substitution also impaired production of virus by cells transfected with JFH1 RNA. This mutation did not alter polyprotein processing or genome replication. This defect in virus production could be rescued by expression of wt NS2 *in trans* from an alphavirus replicon. The *trans*-complementing activities of NS2 from genotypes 1a and 2a demonstrated strong preferences for rescue of the homologous genotype. Importantly, the S168A mutation did not alter the association of core or NS5A proteins with host cell lipid droplets, nor prevent the assembly of core into particles with sedimentation and buoyant density properties similar to infectious virus, indicating that NS2 acts subsequent to the involvement of core, NS5A, and NS3 in particle assembly. Second-site mutations in NS2 as well as in NS5A can rescue the defect in virus production imposed by the S168G mutation. In aggregate, these results indicate that NS2 functions *in trans*, in a late-post assembly maturation step, perhaps in concert with NS5A, to confer infectivity to the HCV particle.

## Author Summary

Worldwide, more than 200 million people are chronically infected with hepatitis C virus (HCV) and thus at risk for fatal liver disease. New, virus-specific therapies are under development by the pharmaceutical industry that target aspects of virus entry and genome replication. However, the process of viral assembly and release from the cell remains cloaked in mystery and thus difficult to target for drug development. Here we describe studies that reveal a previously unrecognized role for the nonstructural protein 2 (NS2) of HCV during a late step in this process. We show that mutations in the carboxy-terminal domain of NS2 disrupt a maturation step that is required to convert a viral assembly intermediate to a fully infectious virus particle. Additional genetic evidence suggests that this maturation step occurs in concert with another nonstructural protein, NS5A. This study thus sheds new light on the role of nonstructural proteins in assembly and maturation of infectious HCV particles and suggests new targets for drug development.

## Introduction

Persistent infection with hepatitis C virus (HCV) is a common cause of chronic liver disease, and may lead to clinically severe cirrhosis and/or hepatocellular carcinoma [1],[2]. Over the two decades that have elapsed since its discovery [3], much has been learned about HCV, the proteins it encodes, and their roles in genome replication. However, investigation of the complete viral life cycle became possible only recently with the development of tractable cell culture-based infection systems [4]–[7]. The availability of these systems has accelerated studies of the mechanisms underlying virus assembly and egress as well as cell entry. In addition to the roles expected of the structural proteins (core, E1, E2) in viral assembly, a number of recent studies suggest that nonstructural proteins, such as NS3 and NS5A as well as p7 and NS2, are also critically important in this process [6]–[16].

NS2 is a small membrane-bound protein with one or more trans-membrane domains [17],[18]. Up until recently, the only known function of NS2 has been its role in polyprotein processing as part of the auto-protease that cleaves *in cis* between NS2 and NS3. However, data from several studies that have used cell culture infection systems suggest the specific involvement of NS2 during the assembly of infectious HCV particles. We showed that compensatory mutations accumulating within the N-terminal domain of NS2 following transfection of an inter-genotypic, 1a/2a chimeric viral RNA enhanced the specific infectivity of secreted virus particles [14]. Subsequently, the deletion of NS2 sequence was shown to block infectious virus production by an otherwise viable di-cistronic HCV RNA that no longer required the NS2-NS3 auto-protease for genome amplification [12]. A structure-function analysis of NS2 has also shown that several amino acid residues within the first trans-membrane domain of NS2 are critical for assembly and release of infectious particles [19]. These studies strongly support a role for NS2 during virus assembly. However, the details of that role and how NS2 might act during the production of infectious virus particles remain unknown.

Here, we show that Ala or Gly substitutions of the Ser-168 residue of NS2 impair infectious virus production without affecting HCV RNA replication or *cis*-processing of the virus polyprotein. Other experiments described here reveal that the defect in infectious virus production can be complemented *in trans* by expression of wild-type NS2 protein in a genotype-specific manner, and that this defect in virus production occurs at a step late in the process of viral assembly and egress, after the interaction of core and NS5A with host cell lipid droplets and the formation of rapidly sedimenting, core-protein containing particles.

## Results

### Ala (or Gly) substitutions of NS2 Ser-168 impair production of infectious virus without affecting RNA replication

**Figure 1:**
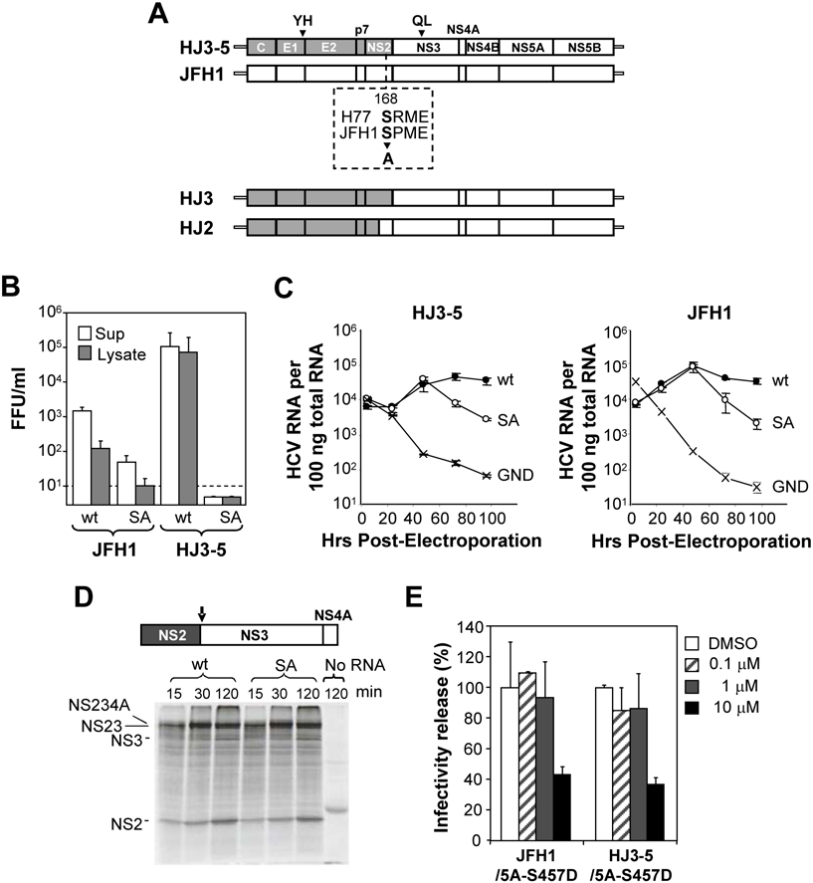
A single mutation in NS2 disrupts infectious virus production without affecting viral RNA replication. (A) Structure of the chimeric HJ3-5 and JFH1 HCV RNAs. HJ3-5 has two compensatory mutations, located within the E1 (YH) and NS3 (QL) sequences from its HJ3 parent [14]. Also shown is the HJ2 chimera, in which the NS2 protein is itself chimeric. The H77 sequence is represented by the shaded boxes, while the JFH1 sequence is open. The location of the putative CK2 phosphorylation site and the S168A mutation within NS2 is highlighted. (B) Infectious virus titers present in supernatant fluids and cell lysates 2 days following transfection of FT3-7 cells with the JFH1 and HJ3-5 RNAs, with (“SA”) or without (“wt”) the S168A mutation. The limit of detection of infectious virus, determined in a replication focus-forming assay (10 FFU/ml), is indicated by the dashed line. (C) Replication of HJ3-5 (left panel) and JFH1 (right panel) RNAs with or without the SA mutation. “GND” represents a mutant genome defective in RNA replication due to the mutation of GDD to GND within NS5B polymerase active site. Viral RNA abundance was measured by quantitative RT-PCR assay of 100 ng of total RNA derived from cell lysates harvested at 4, 24, 48, 72, and 96 h following transfection of the indicated RNA into FT3-7 cells. The data represent a triplicate analysis, ±S.D. (D) Auto-processing of the NS2-NS3 junction site in NS2-NS3-NS4A polyprotein segments translated in vitro in reticulocyte lysates programmed with wt versus the S168A mutant HJ3-5 RNA. The organization of the proteins translated in vitro are shown at the top. Below are shown the protein products of in vitro translation reactions (15–120 min reaction time), separated by SDS-PAGE. There was no difference in the rate of generation of the mature NS2 protein over time in the presence or absence of the SA mutation. (E) Treatment with sub-toxic concentrations of the CK2 inhibitor DMAT does not reduce virus yields from JFH1 or HJ3-5-transfected cells. In addition to the presence or absence of the NS2 SA mutation, the RNAs transfected in this experiment each contained an S457D mutation in NS5A, which eliminates the need for CK2 phosphorylation of this viral protein. The inhibitor was added to the media from 4 to 24 h post-transfection; virus yields were determined by titration of supernatant fluids collected at 48 h.

As indicated above, previous studies suggest that NS2 is likely to have a role in virus assembly and/or release that is distinct from its role during polyprotein processing and genome-amplification as an auto-protease [12], [14], [19]–[22]. Proteins are often modulated by phosphorylation to regulate multiple functions and, since it has been reported that NS2 may be phosphorylated by the host cell kinase CK2 [23], we set out to determine whether NS2 phosphorylation influences the production of infectious HCV particles in cell culture. To this end, we mutated the Ser-168 residue in NS2, which is putatively targeted by CK2 for phosphorylation [23], in two different infectious HCV cDNA clones, one derived from the genotype 2a JFH1 virus, and the other a chimeric genome (HJ3-5) which is comprised of the core-NS2 sequence of the genotype 1a H77 virus in the background of JFH1 with compensatory mutations in E1 (E1-Y170H) and NS3 (NS3-Q221L) [14],[16]. The resulting mutants were named JFH1(SA) and HJ3-5(SA), respectively (Figure 1A). Synthetic RNA derived from either of the parental cDNA clones efficiently produces infectious HCV following transfection into human hepatoma cells (FT3-7 cells, a Huh7 sub-clone). However, the S168A mutation completely abolished infectious virus production by the chimeric HJ3-5 RNA, and significantly reduced the yield of infectious virus released from cells 48 h post-transfection of JFH1 RNAs (Figure 1B). In contrast to lysates of cells transfected with the parental RNAs, in which we could readily detect infectious virus, only a low titer of infectious virus was present in lysates of cells transfected with JFH1(SA) RNA; there was no infectious virus at all in lysates of cells transfected with HJ3-5(SA) RNA, which encodes the genotype 1a NS2 (Figure 1B). These results indicate that Ser-168 of NS2 is involved in a step in infectious virus production that occurs prior to the release of virus from cells. Similar results were obtained with viral RNAs mutated to encode a Gly residue at position 168 in NS2 (HJ3-5(SG) and JFH1(SG) mutants, respectively) (see below).

The impaired production of infectious virus associated with the S168A mutation was not due to an effect on genome replication, since cells transfected with the S168A mutants accumulated HCV RNA at a rate similar to that of wt genomes over the first 48 h post-transfection (Figure 1C). The abundance of the S168A mutant RNA declined after 48 h in transfected cells. In contrast, the increase in abundance of the wt JFH1 or HJ3-5 RNA was sustained beyond 48 h up to 96 h post transfection. This difference in the kinetic of HCV RNA accumulation over time between the S168A mutant and wt genomes is similar to that we have observed previously between an assembly-defective inter-genotypic chimeric RNA (HJ3, Figure 1A) and its derivatives, such as HJ3-5, which are assembly competent due to a compensatory Q221L mutation in the NS3 helicase [16]. Consistent with these results, the S168A mutation did not impair polyprotein processing at the NS2-NS3 junction. This was assessed by determining the self-cleavage activity of an NS2-NS3-NS4A segment derived from the HJ3-5 chimera, with or without the S168A mutation, following its translation in vitro in a cell-free system (Figure 1D). Taken together, these results indicate that the S168A mutation results in a defect late in the virus life cycle, involving either the assembly and/or maturation of infectious particles prior to their release from the cell.

As discussed above, the NS2 S168 residue has been suggested to be targeted by the host cell kinase CK2 for phosphorylation [23]. Since the results above indicate that removal of this putative CK2 target site ablates production of infectious virus, we determined whether pharmacological inhibition of CK2 would similarly prevent the release of infectious virus from cells transfected with viral RNA. However, since CK2 phosphorylation of NS5A Ser-457 is essential for infectious virus production [8], it was necessary to first eliminate the requirement for CK2 phosphorylation at this site. To accomplish this, we introduced an NS5A Ser-457 to Asp mutation in both the HJ3-5 and JFH1 genomes. This mutation has been shown to render the production of virus from a chimeric genotype 2a RNA insensitive to pharmacologic inhibition of CK2, or siRNA knockdown of CK2 expression [8]. These mutated RNAs, HJ3-5/5A-S457D and JFH1/5A-S457D were transfected into FT3-7 cells, which were then treated with a range of concentrations of the CK2 inhibitor, DMAT (2-dimethylamino-4,5,6,7-tetrabromo-1H-benzimidazole), from 4 to 48 h post-transfection. As shown in Figure 1E, DMAT treatment up to 1 µM concentration had no effect on release of infectious virus by either RNA. While we observed a >50% reduction of virus yield by cells treated with 10 µm DMAT, this concentration of the inhibitor causes nonspecific cytotoxicity [8]. These results are consistent with previous findings by Tellinghuisen et al. [8]. They suggest that CK2-mediated phosphorylation of NS2 Ser-168 residue is not essential for the production of infectious virus, and that there is an alternative explanation for the impact of the S168A (or S168G) mutation on this process.

### Expression of NS2 from VEE vectors reveals a genotype-specific difference in NS2 stability that is partially reversed by MG132 inhibition of the proteasome

**Figure 2:**
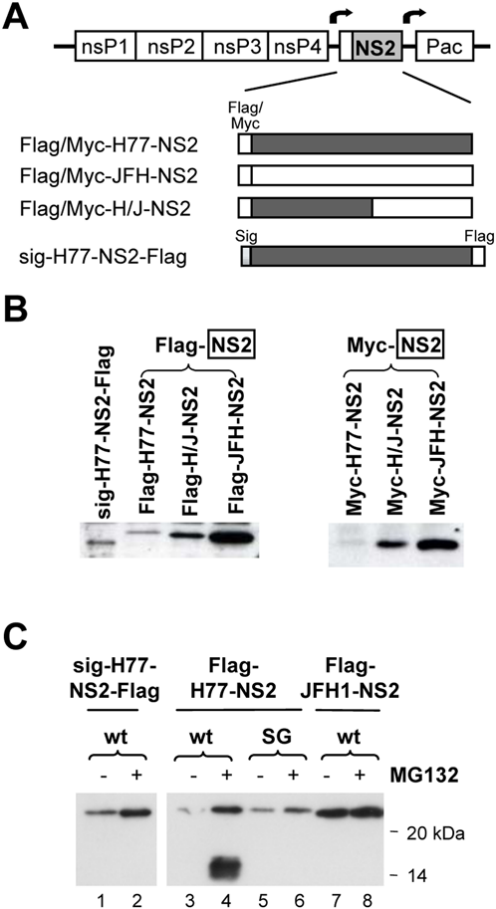
Stability of NS2 proteins expressed by subgenomic VEE replicons. (A) At the top is shown the organization of subgenomic VEE replicons expressing the H77, JFH1, and chimeric HJ2 (“H/J”) NS2 protein. Arrows indicate the VEE subgenomic promoter, while “Pac” indicates the puromycin resistance gene. Shown below are the different NS2 proteins expressed by this series of replicons; each contains either an N- or C-terminal Flag (or Myc) tag. (B) Immunoblot detection of NS2 protein using anti-Flag (left panel) or anti-Myc (right panel) antibodies. With the exception of sig-H77-NS2-Flag, which has a Flag tag at the C-terminal end of the NS2 protein, all of the other NS2 proteins have a Flag or Myc tag at the N-terminus. (C) Treatment with the proteasome inhibitor MG132 enhances the abundance of H77 NS2 (both wt and to a lesser extent the S168G mutant, “SG”) but not JFH1 NS2 expressed by VEE replicon cell lines.

To characterize this defect in virus assembly, we designed a series of experiments aimed at determining whether the S168A assembly defect can be complemented *in trans* by wt NS2 expressed from an alphavirus replicon. Toward that end, we constructed subgenomic Venezualan equine encephalitis virus (VEE) replicons with dual sub-genomic promoters that mediate the expression of NS2 and drug selection marker puromycin N-acetyltransferase (PAC) [24]. Several different replicons were constructed, including replicons expressing the genotype 1a H77 NS2 (Flag-H77-NS2), genotype 2a JFH1 NS2 (Flag-JFH1-NS2), and a chimeric NS2 (Flag-H/J-NS2) comprising N-terminal H77 sequence and C-terminal JFH1 sequence fused at a site of natural recombination [14],[16],[25] (Figure 2). These NS2 proteins were fused to a Flag or Myc tag at their N-terminus, while a fourth construct, sig-H77-NS2-Flag, included the upstream NS2 signal sequence (sig) and was fused to a Flag tag at its C-terminus. We also constructed similar replicons containing the S168A or S168G mutations in NS2. FT7-3 cells were transfected with each of the VEE replicon RNAs, and stable cell lines were selected in the presence of puromycin.

Interestingly, immunoblot analyses of extracts from VEE replicon cells suggested that there is an intrinsic difference in the stability of the wt H77 and JFH1 NS2 proteins. In multiple attempts to establish stable VEE replicon cell lines, the abundance of JFH1 NS2 was always greatest, followed by the chimeric H/J-NS2, and then H77 NS2 (Figure 2B). Similar results were obtained whether the NS2 proteins were fused to either a Flag or Myc tag at the N-terminus. Franck *et al.* [23] reported previously that the NS2 protein expressed by the genotype 1a HCV-H strain (which is closely related to the H77 virus) is unstable, and that its abundance could be increased by treatment with the proteasome inhibitor, MG132. This suggests that the genotype 1a NS2 protein may be degraded in a proteasome-dependent fashion. Franck *et al.* [23] also showed that the S168A mutation enhances the stability of HCV-H NS2. Consistent with this, we observed that the S168G mutation modestly increased the abundance of the H77 NS2 protein expressed from VEE replicons (Figure 2C, compare lanes 3 versus 5). MG132 treatment of VEE replicon cell lines significantly enhanced the abundance of the wt H77 NS2 and, to a lesser extent, the related S168G mutant (lanes 3 versus 4 and 5 versus 6). This was the case with both N- and C-terminally Flag-tagged proteins, whether or not the upstream signal sequence was expressed with NS2 (lanes 1 versus 2). However, it had no effect on the abundance of the JFH1 NS2 protein (lanes 7 versus 8). Inclusion of the S168G mutation also did not enhance JFH1 NS2 abundance (data not shown).

Interestingly, MG132 treatment resulted in the appearance of a ∼14 kDa NS2 degradation product in cells containing the wt Flag-H77-NS2 replicon, while this was not observed with cells expressing wt JFH1 NS2 (lanes 4 versus 8). The H77 NS2 degradation product was derived from the N-terminus of NS2, since it was detected in Flag immunoblots only when Flag was fused at the N-terminus of NS2 (Flag-H77-NS2) and not the C-terminus (sig-H77-NS2-Flag) (lanes 4 versus 2). Moreover, it was not detected in MG132-treated cells expressing the H77 S168G mutant, Flag-H77-NS2(SG) (lanes 4 versus 6). A similar degradation product was not detected in cells expressing wt JFH1 NS2 upon MG132 treatment (lane 8). These results suggest that the H77 NS2 protein is intrinsically less stable than JFH1-NS2, probably because it is more prone to proteasome-mediated degradation. They also suggest the possibility that H77-NS2 undergoes a cleavage event leading to a ∼14 kDa fragment that is degraded by the proteasome. Importantly, the S168G mutation appears to prevent that cleavage and stabilize the H77 NS2 protein. While pulse-chase experiments and experiments to determine whether the H77 NS2 molecule is ubiquitinated are ongoing, these results indicate important differences in stability of the H77 and JFH1 NS2 proteins that may in part explain the difference in the impact of the S168A mutation on infectious virus production by the JFH1(SA) and HJ3-5(SA) RNAs shown in Figure 1B.

### Wild-type NS2 *trans*-complements the S168A defect in virus production in a genotype-specific manner

**Figure 3:**
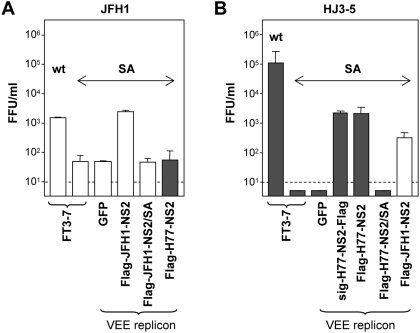
Genotype-specific *trans-*complementation of the NS2 S168A-mediated defect in infectious virus production. (A) Extracellular virus yields (FFU/ml) determined three days after transfection of JFH1 RNA, with or without the S168A mutation (“SA”), into normal FT3-7 cells or VEE replicon cells expressing GFP, Flag-JFH1-NS2, Flag-JFH1-NS2/SA, or Flag-H77-NS2. (B) Extracellular virus yields (FFU/ml) determined in a similar fashion following transfection of the HJ3-5 RNA with or without the S168A mutation into normal FT3-7 cells, or VEE replicon cells expressing GFP, the homologous NS2 sequence with or without the SA mutation, or the heterologous wild-type NS2 sequence (sig-H77-NS2-Flag, Flag-H77-NS2, Flag-H77-NS2/SA, or Flag-JFH1-NS2 replicon cells).

To ascertain the potential for *trans*-complementation of the S168A-imposed defect in virus assembly, we transfected the JFH1(SA) and HJ3-5(SA) RNAs into the NS2-expressing cell lines shown in Figure 2 and monitored release of infectious virus into the extracellular milieu. The presence of a replicating VEE replicon had no effect by itself on the assembly-defective phenotype of the S168A JFH1 mutant, as there was no difference in the extracellular virus yield when the JFH1(SA)-mutant RNA was transfected into the parental FT3-7 cells or cells containing a control, green fluorescent protein (GFP)-expressing VEE replicon (VEE-GFP) (Figure 3A). When JFH1(SA) RNA was transfected into cells expressing Flag-JFH-NS2, the JFH1 infectious virus yield was increased over 10-fold compared to normal FT3-7 cells and restored to the level observed in cells transfected with wt JFH1 RNA (Figure 3A). Transfection of the JFH1(SA) mutant into replicon cells expressing the wt H77 NS2 resulted in no increase in infectious virus production (Figure 3A). Expression of either the N- or C-terminal Flag tagged versions of H77 NS2 rescued the production of infectious virus by cells transfected with HJ3-5(SA) RNA, resulting in a greater than 100-fold increase in infectious virus yields (Figure 3B). Although VEE replicon cells expressing JFH-NS2 also promoted virus production by the HJ3-5(SA) RNA, the virus yield was only 10% that obtained in the Flag-H77-NS2 expressing cells, even though Flag-JFH-NS2 protein expression was much higher (Figures 2 and 3B). VEE replicon cells expressing JFH1-NS2(SA) or H77-NS2(SA) were unable to support the production of virus by JFH1(SA) or HJ3-5(SA) RNA, respectively, confirming that functionally active NS2 protein is required for the *trans*-complementation of virus production (Figure 3A and 3B). In aggregate, these results indicate that expression of wt NS2 is capable of *trans*-complementing an NS2-specific defects, and that this *trans*-complementation activity is genotype-, or possibly strain-specific.

### A compensatory mutation in the N-terminal region of the chimeric H/J-NS2 enhances infectious virus production *in trans*

**Figure 4:**
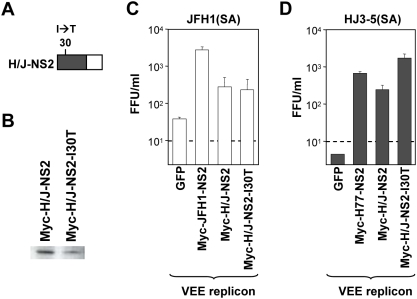
The I30T mutation in NS2 enhances the efficiency of *trans* complementation in a genotype-specific manner. (A) Organization of the chimeric H/J-NS2 replicon expression product, showing the location of the compensatory I30T mutation in the H77-derived sequence [14],[36]. See also Figure 2A. (B) Immunoblot detection of NS2 protein using anti-Myc antibodies. (C,D) Infectious virus yields after transfection of (C) JFH1(SA) or (D) HJ3-5(SA) RNA into VEE replicon cells expressing GFP, or Myc-H/J-NS2 with or without the I30T mutation. Extracellular virus yields were determined three days following transfection. The detection limit of the infectivity assay is indicated by the broken line.

We next assessed the ability of the chimeric H/J-NS2 protein to *trans*-complement the virus assembly defect in the JFH1(SA) and HJ3-5(SA) mutants. As shown in Figure 4C and 4D, cells expressing the chimeric (H/J)-NS2 were capable of supporting the production of infectious virus by either JFH1(SA) or HJ3-5(SA) RNA,although to a lesser extent than cells expressing the homologous JFH1 or H77 NS2.

We previously reported that several naturally evolving compensatory mutations in the N-terminal domain of NS2 enhanced the production of infectious virus (but not viral genome replication) by a chimeric HCV genome (H-(NS2)-J RNA, referred to herein as HJ2) [14], in which sequence encoding the structural proteins of the genotype 1a H77 virus had been placed within the context of the JFH1 genome, and in which the NS2 protein was identical to the H/J-NS2 chimera expressed by the VEE replicons (Figure 1A). To determine whether the positive effect of these compensatory NS2 mutations could be re-created in the *trans*-complementation system, we constructed a VEE replicon expressing H/J-NS2 containing one such mutation, NS2 I30T (I839T in the H77 polyprotein sequence). Cells expressing this mutated chimeric NS2 (Myc-H/J-NS2-I30T) produced a 6- to 8-fold higher yield of infectious virus when transfected with HJ3-5(SA) RNA, compared to cells expressing the chimeric NS2 without the compensatory mutation (Figure 4D). Significantly, the presence of this mutation had no effect on the efficiency of virus production in cells transfected with the JFH1(SA) RNA (Figure 4C), again indicating the genotype- (or strain-)specific nature of *trans*-complementation.

In aggregate, these data indicate that both the N-terminal and C-terminal domains of NS2 are somehow involved in processes required for production of infectious virus particles. Of note, however, is that our prior work suggests that the NS2 I30T mutation does not influence assembly *per se*, but rather enhances the specific infectivity of released virus particles, suggesting an effect on a late, post-assembly maturation step [14].

### The C-terminal domain NS2 is essential for *trans*-complementation of virus production

**Figure 5:**
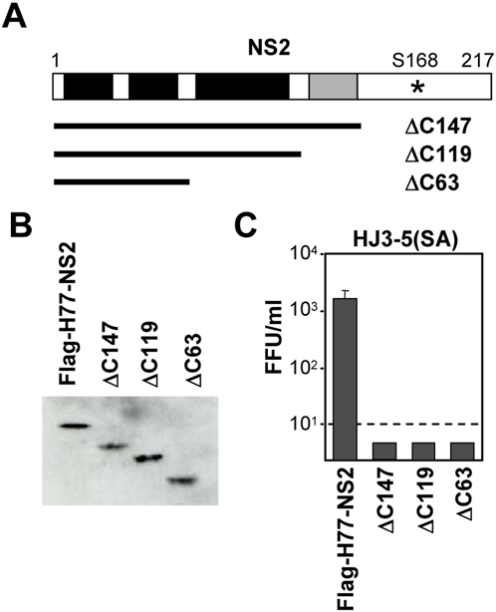
C-terminal deletion mutants of NS2 are incapable of complementing the S168A defect in virus production. (A) NS2 C-terminal deletion mutants expressed by modified VEE-Flag-H77-NS2 replicons. The black and grey shaded areas indicate four potential transmembrane domains within NS2 that have been reported previously [18]. The grey sequence area needs to be located within the cytoplasm to function as part of the NS2-NS3 protease [20]. The position of Ser-168 is indicated. (B) Immunoblots of full-length and C terminally truncated versions of the H77 NS2 protein expressed by the VEE replicon cell lines. (C) Extracellular infectious virus yields determined three days after transfection of the mutant HJ3-5(SA) RNA into the VEE replicon cell lines expressing wt and C-terminal deletion mutants of H77 NS2.

To better define the NS2 domains required for *trans*-complementation of S168A-mediated defect in virus production, we constructed a series of VEE replicons expressing H77-NS2 protein mutants with N- and C-terminal deletions of increasing length. Most of the N-terminal deletion mutants were unstable, and their expression could not be detected by immunoblotting or immunofluorescence microscopy (even with MG132 treatment) in cells transfected with the replicons (data not shown). However, the C-terminal deletion mutants were expressed at an abundance similar to that of the wt protein (Figure 5A and 5B). Nonetheless, when the HJ3-5(SA) mutant RNA was transfected into VEE replicon cells expressing these C-terminal deletion mutants, none were capable of trans-complementing the S168A defect in virus production. These data indicate that NS2 contains an essential domain within its C-terminal 71 residues that is required for production of infectious virus particles, consistent with the phenotype of the S168A and S168G mutants.

### The NS2 S168G mutation does not prevent assembly of fast-sedimenting core-containing particles, suggesting a defect late in infectious virus production

As described previously, the chimeric HJ3-5 RNA used in the experiments described above does not produce infectious virus particles in the absence of one or more compensatory mutations within the NS3 helicase domain (Q221L in HJ3-5, Figure 1A). These NS3 mutations are required for the intracellular assembly of core protein-containing particles that have the sedimentation properties of infectious virions [14],[16]. The ability of these mutations to compensate for the assembly defect in the parental chimeric RNA (H-(NS2/NS3)-J, referred to herein as HJ3) (Figure 1A), has thus defined an important role for NS3 during an early step in infectious virus production that involves particle assembly [14],[16]. To ascertain whether the Ala or Gly substitution at Ser-168 of NS2 causes a similar early defect in particle assembly, or acts at a later, post-assembly step, we characterized the sedimentation properties of core protein present in extracts of HJ3-5 and HJ3-5(SG) RNA transfected cells. This was accomplished by rate-zonal centrifugation of cell extracts in sucrose gradients, followed by immunoblotting individual gradient fractions to detect the presence of the core protein. These experiments were carried out in cells containing the GFP- or H77-NS2-expressing VEE replicon. Results were compared with those from cells transfected with the assembly-defective HJ3 chimeric RNA as a control.

**Figure 6:**
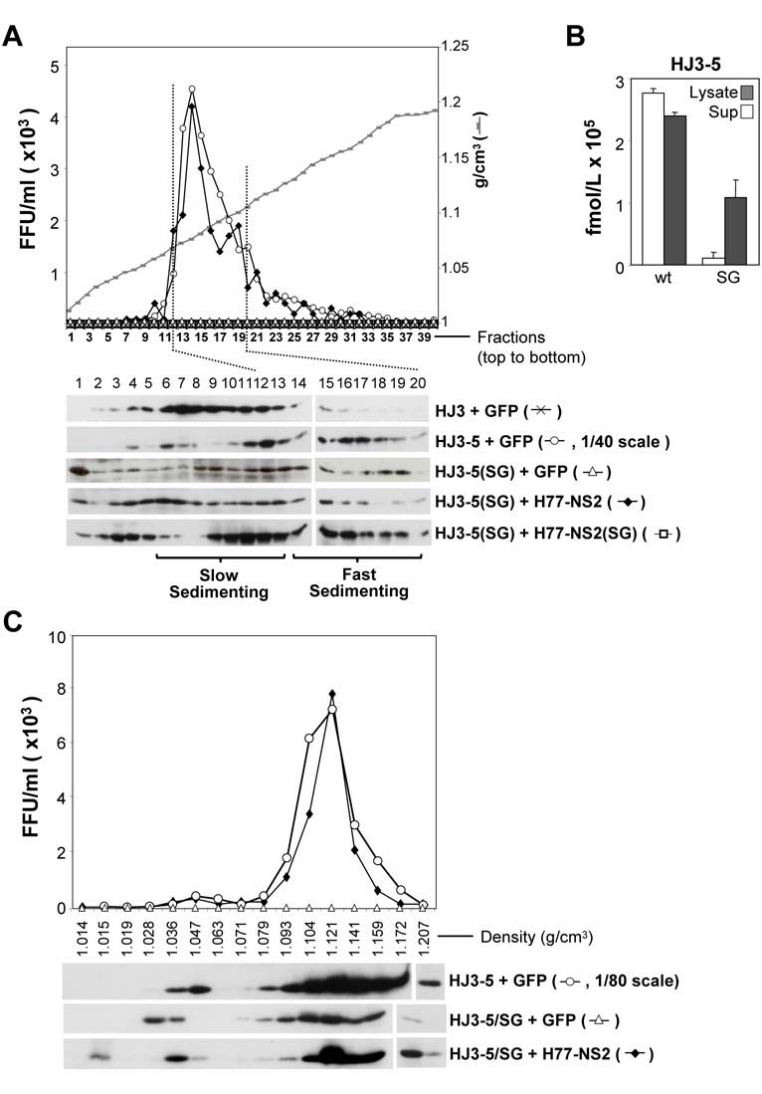
Detection of fast-sedimenting, core-containing HJ3-5(SG) particles with physical properties similar to infectious virus. (A) Rate zonal centrifugation of cell lysates derived from VEE replicon cell lines expressing GFP, Flag-H77-NS2, or Flag-H77-NS2(SG) following transfection with the HJ3, HJ3-5, and HJ3-5(SG) RNA. A total of 40 fractions were collected from each gradient: the titer of infectious virus in fractions from HJ3-5 transfected cells was reduced in scale 40-fold to allow it to be plotted on the same graph as the *trans*-complemented infectious particles present in lysates of Flag-H77-NS2 cells transfected with the mutant HJ3-5(SG) RNA. Cell lysates were prepared three days following transfection by multiple rounds of freeze-thawing. The top panel shows the sedimentation profile of infectious HJ3-5 and trans-complemented HJ3-5(SG)-derived particles. The bottom panel shows immunoblot detection of core protein in fractions 1–20 of the gradients. The fractions containing the peak infectivity are indicated with dashed lines. Slow-sedimenting core antigen was present in fractions 6–13, while fast-sedimenting particles were found in fractions 14–19. (B) Core antigen measured by a quantitative ELISA assay in cell lysates or supernatant fluid from FT3-7 cells transfected with HJ3-5 RNA, with or without the S168G mutation. (C) Equilibrium density gradient ultracentrifugation of particles present in VEE replicon cell lysates expressing GFP or Flag-H77-NS2, three days post-transfection of the HJ3-5 or HJ3-5(SG) RNA. Infectious virus titer of each fraction is shown in the top panel, with the scale reduced 80-fold for HJ3-5 to allow it to be plotted in the same graph as the trans-complemented HJ3-5(SG) –derived virus. The relative distribution of core antigen present in each gradient fraction, as determined by immunoblotting, is shown in the lower panel.

Results from this series of experiments are shown in Figure 6A. Consistent with previously reported data [16], peak core protein abundance was found in fractions 6–12 (from the top) of gradients loaded with extracts of HJ3-transfected cells (“slow sedimenting”) (Figure 6A, lower panel). In contrast, the bulk of the core protein present in lysates of cells transfected with HJ3-5 (which contains the compensatory Q221L mutation) sedimented more rapidly, and was present in gradient fractions 12–19 (“fast sedimenting”) [16]. These latter fractions also contained the peak HJ3-5 infectious virus titer (HJ3 produces no infectious virus) (Figure 6A, upper panel). Importantly, although some core was found at the very top of the gradient (fraction 1), a significant portion of the core protein present in lysates of GFP-expressing cells transfected with HJ3-5(SG) RNA was also present in fractions 12–19 (Figure 6A). This result indicates that the S168G mutation does not prevent the assembly of fast-sedimenting virus-like particles, despite the absence of infectious virus production. Thus, the S168G mutant appears to be defective in a late, post particle-assembly step, in virus production.

Despite the assembly of core into fast-sedimenting intracellular particles, we were not able to detect the release of a measurable amount of core protein from cells transfected with the HJ3-5(SG) mutant using a sensitive ELISA assay (Figure 6B). In contrast, core protein was readily detected in supernatant media from cells transfected with the parental HJ3-5 RNA (Figure 6B). Taken together with the results shown in Figure 6A, these results suggest that at least one of the functions of NS2 protein is to confer infectivity to pre-assembled core containing particles, and that in the absence of this, these “fast sedimenting” non-infectious particles are prevented from being released from the cell. Consistent with this interpretation, the amount of core protein present in lysates of cells transfected with the S168G mutant was over 10-fold the limit of detection in the ELISA assay, and thus at least 10-fold greater than the abundance in the supernatant fluids (Figure 6B). In contrast, the concentration of core was slightly greater in the supernatant fluids than in extracts from the HJ3-5 transfected cells. Gastaminza *et al.* [26] recently demonstrated that high density, infectious, intracellular HCV particles are prone to degradation in a proteasome-independent manner unless they undergo a maturation step that is necessary for secretion [26]. The data shown in Figure 6A and 6B are consistent with this model for virus production, and suggest that NS2 plays a key role in the transition from non-infectious, intracellular particles to infectious particles capable of secretion.

When we similarly analyzed lysates of H77-NS2 expressing replicon cells transfected with the HJ3-5(SG), we found that the infectious virus produced by *trans*-complementation banded within fractions 12–19, similar to virus produced by the parental HJ3-5 RNA in GFP-expressing cells (Figure 6A, upper panel). Consistent with these results, a significant proportion of the core protein was also detected in these fractions (Figure 6A, lower panel). However, a substantial amount of the core protein also sedimented slowly (fractions 3–11), consistent with the relatively low efficiency of *trans*-complementation and the 40-fold lower titer of infectious virus produced compared with wt HJ3-5 RNA (see legend to Figure 6A). We observed similar results, including a substantial proportion of core protein within fractions 12–19, when we analyzed lysates of VEE H77-NS2(SG) replicon cells transfected with the HJ3-5(SG) RNA (Figure 6A, bottom panel). These results provide compelling evidence that the NS2 S168G mutation does not prevent the assembly of rapidly-sedimenting, core-containing particles despite the inability of RNA bearing this mutation to produce infectious virus. While the distribution of the rapidly sedimenting core antigen associated with the noninfectious particles that formed in the HJ3-5(SG) RNA-transfected cells across fractions 12–19 of these gradients was very similar to the distribution of core associated with the infectious particles formed in cells transfected with the assembly-competent HJ3-5 RNA, these experiments do not exclude significant differences in the composition of these particles.

To further characterize the non-infectious and *trans*-complemented, infectious HJ3-5(SA) particles, we ascertained their buoyant density by equilibrium centrifugation of cell lysates in iodixanol density gradients and compared these results with intracellular HJ3-5 particles. As shown in Figure 6C, the densities of the infectious intracellular particles derived from the HJ3-5 and *trans*-complemented HJ3-5(SG) RNA were indistinguishable, with peak infectivity for each banding between 1.104 and 1.121 g/cm^3^. Most of the core protein present in these lysates banded at the same density as infectious particles, while lesser amounts were found in less dense fractions (1.028–1.047 g/cm^3^) (Figure 6C). Very similar results were obtained in immunoblots of fractions from gradients loaded with the non-infectious HJ3-5(SG) particles. These results indicate that the buoyant density of the non-infectious HJ3-5(SG) particle is similar to the infectious particles produced by *trans*-complemented HJ3-5(SG) RNA and the parental HJ3-5 genome, despite the absence of infectivity.

### The NS2 S168G mutation does not alter the co-localization of core and NS5A on lipid droplets

**Figure 7:**
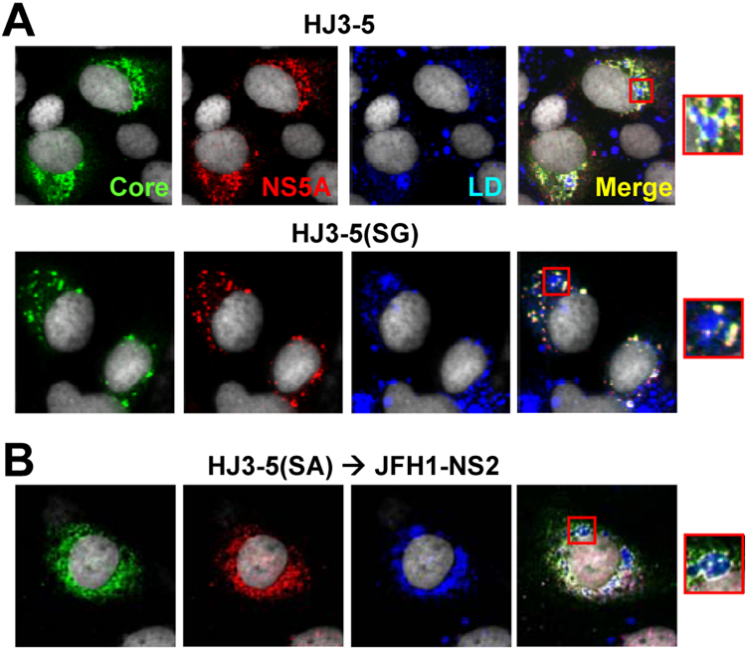
Mutation of NS2 S168 does not perturb the co-localization of core and NS5A on the lipid droplet. (A) FT3-7 cells were transfected with HJ3-5 or HJ3-5(SG) RNA, and two days later washed and fixed, as described in Materials and Methods, then labelled with antibodies specific for core protein (green) and NS5A (red) followed by examination with a Zeiss LSM 510 Meta microscope. Neutral lipid staining is shown in blue. At the right is an enlarged area from the merged image showing enlarged views of solitary lipid droplets (see box). (B) HJ3-5(SA) RNA was transfected into the VEE replicon cell line expressing FlagJFH1-NS2. The intracellular localization of core and NS5A was determined as described in (A).

Recent reports indicate that the NS5A protein plays a critical role in the assembly of HCV particles by recruiting replication complexes to core protein localized on the surface of lipid droplets where particle assembly takes place [9],[10],[27]. Since the results shown in Figure 6 suggest that the mutations in Ser-168 of NS2 impact a later, post-assembly step in the production of infectious virus, we considered it likely that these mutations would not interfere with the association of NS5A with the core protein on lipid droplet, despite the ability of the mutations to ablate infectious virus production from HJ3-5 RNA. This was confirmed by laser-scanning confocal microscopy of FT3-7 cells transfected with the HJ3-5 or HJ3-5(SG) RNAs, which revealed co-localization of core and NS5A protein on lipid droplets in cells transfected with either RNA (Figure 7A). Thus, the NS2-S168G mutation has no impact on the interaction of core with NS5A on lipid droplets. This is consistent with the rate-zonal gradient analysis shown in Figure 6A, which indicates that replication of the HJ3-5(SG) RNA leads to the assembly of core protein-containing particles with sedimentation properties similar to the infectious virus particles produced from the wt genome. The colocalization of core and NS2 on lipid droplets was unchanged when the HJ3-5(SA) mutant RNA was transfected into cells containing a replicon expressing JFH1-NS2 (Figure 7B).

### NS2 co-localizes strongly with E2, and partially with NS5A, but not with core

**Figure 8:**
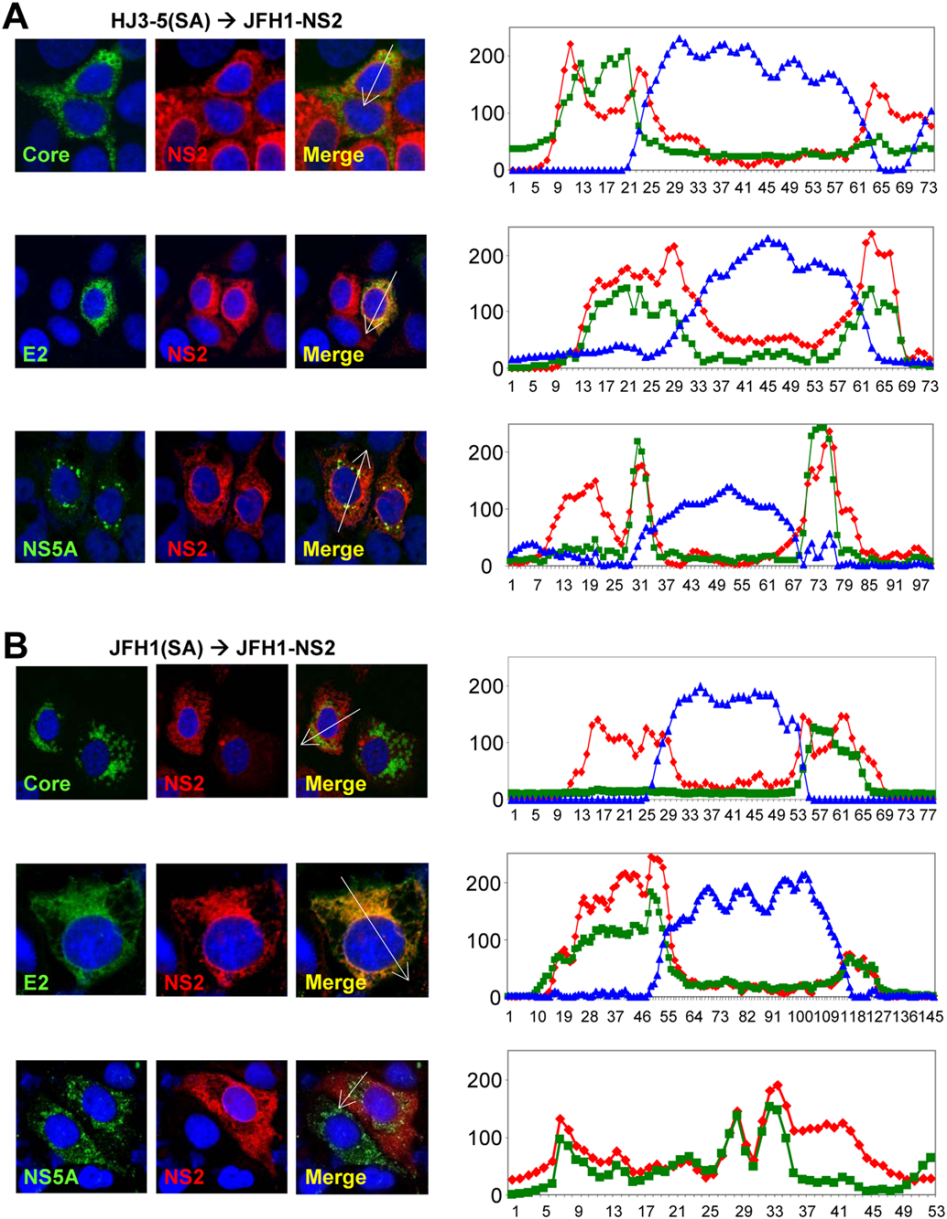
Confocal microscopic imaging of intracellular NS2 distribution. NS2 protein co-localizes with E2 but not core protein. VEE replicon cells expressing FlagJFH1-NS2 were transfected with (A) HJ3-5(SA) or (B) JFH1(SA) RNA and fixed and labeled two days later with antibodies specific for core protein (green, upper panel), E2 (green, middle panel), NS5A (green, lower panel), or NS2 (red). Slides were subsequently examined with a Zeiss LSM 510 Meta microscope. At the right are line scan results obtained using MetaMorph software depicting a linear trace of the fluorescence intensity of individual pixels along a segment of the white arrow overlaying the merged image. The Y axis represents average pixel intensity while the X axis represents distance from the origin. Red, green, and blue traces correspond to the color of the fluorophores shown in merged images. This analysis revealed a high level of colocalization between NS2 and E2, but only partial colocalization of NS2 and NS5A.

Confocal microscopy also demonstrated that the Flag-JFH1-NS2 protein expressed from VEE replicons was distributed in a reticular pattern throughout the cytoplasm, but with particularly strong staining near the nuclear membrane (Figure 8A and 8B). Flag-H77-NS2 showed a similar distribution (data not shown), but a much lower signal intensity consistent with lower protein abundance determined in immunoblots (Figure 2). Since an assembly-defective core mutant was recently shown to be rescued by a second-site mutation within NS2 [15], we used confocal microscopy to determine whether there was co-localization of these two proteins in VEE Flag-JFH1-NS2 replicon cells transfected with the HJ3-5(SA) RNA. As shown above, the expression of the JFH1 NS2 protein in these cells rescues the ability of the HJ3-5(SA) RNA to produce infectious particles (Figure 3B). However, there was no demonstrable co-localization of NS2 and core in these cells (Figure 8A, upper panels), nor in JFH1 NS2-expressing cells transfected with JFH1(SA) RNA (Figure 8B, upper panel).

The reticular staining pattern observed for NS2 in these studies resembles that of E1 and E2, which localize to the ER membrane [28]. Consistent with this, there was strong co-localization of E2 and NS2 when the Flag-JFH1-NS2 replicon cells were transfected with either HJ3-5(SA) or JFH1(SA) (Figure 8A and 8B, middle panels). This was confirmed by a quantitative pixel analysis of these confocal microscopic images (see the panels to the right in Figure 8A and 8B). Since most E2 protein does not co-localize with core in JFH1 infected cells [28], the co-localization of NS2 with E2 is consistent with the absence of co-localization with core. Quantitative pixel analysis also suggested partial co-localization of NS2 with NS5A in these experiments, but to a lesser extent than with E2 (we used Myc-JFH1-NS2 replicon cells in this experiment, as this allowed simultaneous labeling of NS5A with rabbit antibody and NS2 with murine anti-Myc) (Figure 8A and 8B, lower panels). Taken together, these results suggest that NS2 and E2 localize to membranes of the peri-nuclear ER, distinct from core which strongly localizes to the lipid droplet. NS5A associates with core on the lipid droplet, but also is found in association with NS2, a fact that may be important in particle assembly.

### Second-site mutations in NS2 and NS5A rescue the NS2 S168G-mediated defect in production of infectious virus

To better understand how Ser-168 might function to confer infectivity on pre-assembled HCV particles, we transfected cells with the HJ3-5(SA) RNA and attempted to isolate revertants with second-site mutations capable of rescuing the ability of the HJ3-5(SA) RNA to produce infectious virus. In multiple transfection experiments, infectious virus began to be released from cells after 6–7 cell passages. However, sequencing of RNA extracted from these viruses invariably revealed reversion of the S168A mutation to the wt sequence (a single nucleotide change is sufficient). Different results were obtained with HJ3-5(SG), in which two nucleotide substitutions are required to change the codon (‘GGG’) from Gly back to Ser. We successfully isolated revertants in 4 independent transfection experiments, with release of infectious virus first detected between cell passages 7 to 11 (Table 1). Sequencing of these viruses revealed mutations at Leu-174 of NS2 in 3 out of 4 independent transfection experiments: this residue was changed to Val in 2 experiments, and to a mixture of Leu and Ile in the third experiment, in concert with a mixture of Trp and Arg at Arg-68 in the protease domain of NS3. (The HJ3-5(SG) clone used in these experiments was found subsequently to contain an adventitious NS5A mutation, Thr-115 to Ala; this had partially or completely reverted to the wild-type Thr sequence in virus from each of these 3 transfection experiments). The only mutation found in infectious virus recovered from the fourth transfection experiment was in NS5A, in which Val-464 was changed to Leu. These mutations are summarized in Table 1.

**Table 1:** Mutations identified in infectious HJ3-5(SG) pseudo-revertants.

Protein	Parent Clone	Exp. #1	Exp. #2	Exp. #3	Exp. #4
NS2	Leu-174	Ile*	Val		Val
NS3	Arg-68	Trp*			
NS5A	Ala-115‡	Thr*	Thr		Thr
NS5A	Val-464			Leu	

*: Present as a mixture with parental sequence.

**‡:** Adventitious mutation in the parental clone.

**Figure 9:**
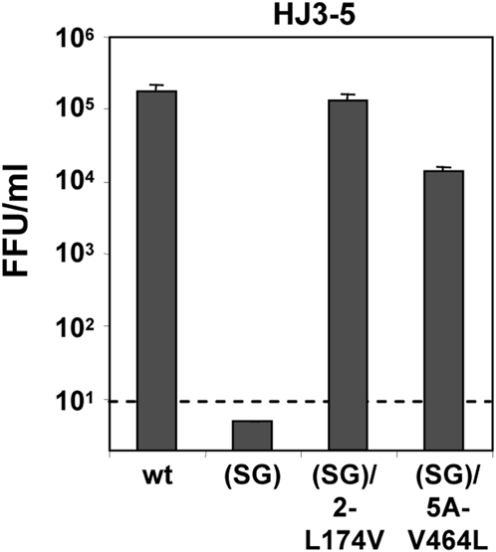
Second-site mutations in NS2 or NS5A rescue infectious virus production by HJ3-5(SG) RNA. Extracellular virus yields were determined two days post-transfection of FT3-7 cells with the indicated RNAs. Results shown represent the average from two independent experiments.

To confirm that these mutations render the HJ3-5(SG) RNA capable of producing infectious virus, we independently introduced the NS2-L174V and NS5A-V464L mutations into the HJ3-5(SG) construct (resulting in HJ3-5(SG)/2-L174V and HJ3-5(SG)/5A-V464L, respectively) and assessed the ability of the modified RNAs to produce infectious virus by measuring infectivity in supernatant fluids 2 days after transfection. The NS2-L174V mutation, which is only 6 residues distant from the S168G mutation in HJ3-5(SG), resulted in robust production of infectious virus, fully restoring the ability of the HJ3-5(SG) RNA to produce infectious virus (Figure 9). Interestingly, the JFH1 NS2 protein already contains Val at residue 174. Therefore, it is tempting to speculate that the lesser inhibition of virus production we observed when we placed the S168A mutation in the JFH1 compared to the HJ3-5 background (Figure 1B) may reflect this natural polymorphism.

The NS5A-V464L mutation also restored the ability of the HJ3-5(SA) RNA to produce infectious virus. However, although the NS5A-V464L mutation enhanced virus production at least 1000-fold, infectious virus yields from the HJ3-5(SG)/5A-V464L RNA were about 10-fold less than with wt HJ3-5 RNA (Figure 9). This genetic interaction between NS2 and NS5A is consistent with a direct physical interaction of the two proteins as suggested by the partial co-localization of NS2 and NS5A detected by confocal microscopy (Figure 8A and 8B, lower panels). Interestingly, the V464L mutation is close to a putative CK2 phosphorylation site in the C-terminal domain of NS5A (Ser-457) that regulates the production of infectious JFH1 virus [8]. It also has been identified during selection of JFH1 variants capable of producing higher yields of infectious virus in cell culture [29].

## Discussion

NS2 is a small trans-membrane protein that is located between the structural and nonstructural proteins within the HCV polyprotein [17],[18]. Until recently, the only known role for this protein in the viral life cycle was its auto-protease activity, which cleaves the polyprotein between NS2 and NS3 [20],[22],[30]. However, along with newly recognized roles for two other nonstructural proteins, notably NS5A [8]–[10] and NS3 [16], in viral particle assembly, there is growing evidence that NS2 also has essential function(s) that are required for the production of infectious virus [12],[14],[15],[19]. In the work described here, we have shown that a highly conserved amino acid residue within NS2 (Ser-168) is essential for the production of infectious virus particles, as the mutation of this residue to Ala or Gly either eliminated or substantially reduced the yield of infectious virus released by cells transfected with viral RNAs containing sequences encoding either genotype 1a or 2a NS2 protein (Figure 1B). Furthermore, we show that the defect in infectious virus production that is caused by mutation of Ser-168 is within a late step in this process, one that occurs subsequent to the assembly of core-protein containing particles, as non-infectious, intracellular particles with sedimentation properties and buoyant densities similar to infectious virus still formed within cells transfected with the mutant RNAs (Figure 6A). Consistent with this, core and NS5A proteins were co-localized, as they normally are, on the surface of lipid droplets in HJ3-5(SG) transfected cells (Figure 7A). Thus, the defect imposed by the Ser-168 mutations appears be within a maturation step that confers infectivity on previously assembled particles. Since the non-infectious, core-containing particles that are assembled by these NS2 mutants are not released from the cell (Figure 6B), this maturation process may be required for their release from the cell. The data presented here thus reveal a novel role for NS2, most likely in cooperation with NS5A (see below), during the maturation of HCV particles late in the process of virus assembly and release.

Our focus on the Ser-168 residue in NS2 was prompted by the report of Franck *et al.* [23], who suggested that this residue (in the genotype 1a HCV-H strain of HCV) is targeted by the host kinase, CK2, for phosphorylation. However, although we found that both S168A and S168G mutations ablated production of infectious virus by HJ3-5 RNA (which contains NS2 sequence from H77) (Figure 1B), the lack of a specific effect of DMAT on production of infectious virus by this RNA strongly suggests that phosphorylation of Ser-168 by CK2 is not required for this process (Figure 1E). These latter data are consistent with recent work by Tellinghuisen et al. [8], who identified a CK2 phosphorylation site in NS5A and reported that treatment with a CK2 inhibitor did not impair infectious virus release by a genotype 2a RNA (J6/JFH1 chimera) provided the NS5A residue targeted by CK2 was first mutated to Asp. Importantly, we found that mutations at NS2 Ser-168 significantly impaired infectious virus production by viral RNAs containing either the genotype 1a (HJ3-5) or 2a (JFH1) NS2A sequence. Taken together, these data suggest that the NS2 Ser-168 mutations are likely to inhibit the production of infectious virus by a mechanism other than interference with CK2 phosphorylation of this residue.

As described by Franck et al. [23], we observed that the genotype 1a H77 NS2 protein was subject to proteasome-mediated degradation (Figure 2). While Franck et al. suggested that CK2 phosphorylation of Ser-168 regulates the stability of the H77 NS2 protein, our data suggest that the protein is partially, but not completely, stabilized by a Gly substitution at this residue (Figure 2C). In contrast, the wt genotype 2a JFH1 NS2 does not appear to be subject to proteasome-mediated degradation (Figure 2C), although it shares the same putative CK2 target site, Ser-168 in NS2, along with other HCV sequences [23]. The difference in the intrinsic stability of the NS2 proteins from these two distinct viral strains may account, at least in part, for the differences we observed in the magnitude of the impact of S168A or S168G mutations on infectious virus production by the HJ3-5 and JFH1 RNAs. The S168A mutation completely abolished infectious virus production by HJ3-5, while causing an incomplete loss of infectious virus yield from the JFH1 RNA (Figure 1B). However, we also observed that the H77 NS2 could *trans*-complement the production defect in HJ3-5(SA) but not JFH1(SA) RNA, while the JFH1 NS2 can rescue virus production by either RNA (although with a greater effect on the JFH1(SA) defect) (Figure 3A and 3B). These differences in NS2 *trans*-complementation cannot be attributed simply to differences in NS2 stability, and suggest more fundamental differences in NS2-protein interactions required for particle maturation. Whether these differences are restricted to these two specific strains of HCV, or instead reflect broader, genotype-specific differences (genotype 1a versus 2a), will require further study.

**Figure 10:**
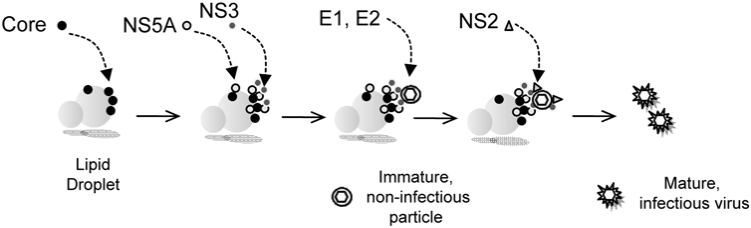
A model for sequential involvement of non-structural proteins in HCV assembly, maturation, and egress. Particle assembly follows the recruitment of core, followed by NS5A and NS3 to the surface of lipid droplets. Such particles are noninfectious, however, and must undergo a maturation step mediated by NS2 (most likely in concert with NS5A) prior to becoming infectious and exiting the cell. See the Discussion for details.

Importantly, second site mutations involving either Leu-174 in NS2 (residue 938 in the polyprotein), or Val-464 in NS5A (residue 2440) were capable of rescuing the defect in virus production mediated by the S168G mutation (Figure 9). An X-ray crystallographic reconstruction of the NS2 auto-protease domain suggests that both Ser-168 and Leu-174 are surface-exposed residues [20]. Therefore, it seems reasonable to speculate that Ser-168 and Leu-174 may be involved in protein-protein interaction(s) required for the maturation of previously assembled particles and leading to the production of infectious virus. The fact that the V464L mutation in NS5A also rescues infectious virus production by the NS2 S168G mutant suggests the possibility that this putative NS2 interaction could involve NS5A (Figure 9). While other explanations are possible for the genetic interaction we observed between NS2 and NS5A, a close physical interaction is suggested by the partial co-localization of NS2 and NS5A identified by confocal microscopy (Figure 8). Whether or not a direct interaction occurs between NS2 and NS5A, the rescue of the late NS2 defect in virus production by a second-site mutation in NS5A indicates that NS5A acts at multiple, temporally distinct points in the production of infectious virus particles. First, in an early step in virus assembly, NS5A is recruited to the core protein which decorates the surface of lipid droplets [8]–[10]. This is a pre-requisite for particle assembly, but it is not sufficient, as we have recently described an NS3 defect in infectious virus production that ablates intracellular particle assembly but does not impair the association of NS5A with core on lipid droplets [16]. The data we have presented in this paper indicate that NS2 acts at a later step in the process of infectious particle production, in association with NS5A but subsequent to the earlier involvement of core, NS5A and NS3, to confer infectivity on previously assembled particles in a maturation step that appears to be required for release of virus to the extracellular environment. This hypothetical sequence for the contribution of individual nonstructural proteins to infectious particle assembly and release is summarized in Figure 10.

HCV is classified within the family Flaviviridae, and thus it is related phylogenetically to yellow fever virus (YFV) as well as Kunjin virus. It is interesting to note that the NS2A proteins of YFV and Kunjin have also been implicated in virus assembly and release, and in both cases this function can be *trans*-complemented by expression of the wt protein [31],[32], as we have shown here for HCV. Although the flavivirus NS2A proteins differ substantially from the NS2 protein of HCV both in sequence and functions within the viral life cycle, it is not unreasonable to suspect that they may share some mechanistic features in common during the process of virus assembly and release. In the case of the Kunjin NS2A protein, its role in infectious virus assembly and release appears to be closely linked to the production of specific virus-induced membrane alterations [32]. We observed very strong co-localization of the HCV NS2 protein with the major envelope protein, E2, in VEE replicon cells during infectious virus production (Figure 8A and 8B, middle panels). E2 and NS2 were both prominently localized to membranes of the peri-nuclear ER, suggesting the possibility of an interaction between NS2 and E2 during the maturation process. NS2 may interact with E1 as well, since we showed recently that a mutation in E1 (Y170H) functions cooperatively with a mutation in NS2 (I30T), but not P7 (Y31C), to enhance the infectious yield of virus produced from a genotype 1a/2a chimera ([14] and Yi M, Lemon SM, unpublished data). One possibility is that NS2 might facilitate a rearrangement of the envelope proteins following assembly of the particle that confers or enhances infectivity. Such an envelope rearrangement would not be without precedent among the flaviviruses, and could be required for infectivity of the HCV particle. Additional studies of the HCV assembly and release process will be required to assess this possibility, as well as a detailed characterization of the structure of the infectious virus particle.

## Materials and Methods

### Plasmids

The parental and chimeric HCV plasmids used in these studies have been described previously: pHJ3-5 (pH-(NS2/NS3)-J/YH/QL) was derived from pHJ3 (pH-(NS2/NS3)-J) by introducing two compensatory mutations, one located within E1 (Y361H) and the other in NS3 (Q1251L), that allow for assembly of infectious virus particles following transfection of Huh7 cells [14],[16]. The NS2 S168A mutation was introduced into the pHJ3-5 and pJFH1 plasmids resulting in pHJ3-5(SA) and pJFH1(SA) by QuikChange mutagenesis (Stratagene) and confirmed by sequencing analysis. Similar methods were utilized to construct and validate the NS2 S168G and the various NS5A mutants described in the text. VEE replicons expressing various NS2 proteins were constructed using p5′VEErep/L/GFP/Pac [24], a generous gift from Dr. Ilya Frolov (University of Texas Medical Branch at Galveston). To facilitate these constructions, a *Pac*I site was introduced at the end of the GFP coding sequence in this plasmid using QuikChange mutagenesis (p5′VEErep/L/GFP/Pac v.2). NS2 coding sequence was amplified from pH77S or pJFH1 by PCR using primers that were designed to introduce *Xba*I and *Pac*I sites at the 5′- and 3′-termini of the NS2 sequence. Additional sequences encoding Flag or Myc tags were placed at the N- or C-terminal end of NS2, as shown in Figure 2A. The NS2 fragments were digested with *Xba*I and *Pac*I before being ligated to two DNA fragments (*Pac*I/*Mlu*I and *Mlu*I*/Xba*I) derived from p5′VEErep/L/GFP/Pac v.2, resulting in the NS2-expressing VEE replicon constructs. The NS2-S168A and S168G mutations were introduced subsequently by QuikChange mutagenesis. To introduce the NS2-L174V mutation, HJ3-5 was digested with *Bgl*II/*Mlu*I and ligated to a PCR fragment containing both NS2-S168G and L174V mutations. C-terminal NS2 deletion mutants ΔC63, ΔC119, ΔC147 were constructed using QuikChange mutagenesis. The integrity of the constructs was validated by DNA sequencing of the manipulated regions.

### Cells

FT3-7 and Huh-7.5 cells are clonal derivatives of Huh7 human hepatoma cells [14],[33]. They were grown in DMEM containing 10% FBS and 1× penicillin/streptomycin at 37°C in a 5% CO_2_ environment. VEE replicon cells were cultured in the same medium plus 1 µg/ml puromycin (Invitrogen).

### HCV RNA transfection and virus production

HCV RNAs were transcribed in vitro and electroporated into cells as described previously [34]. In brief, for electroporation,10 µg of in vitro-synthesized HCV RNA was mixed with 5×10^6^ FT3-7 cells in a 2-mm cuvette and pulsed twice at 1.4 kV and 25 µF in a Gene Pulser II (BioRad) apparatus. Cells were subsequently seeded into 12-well plates for analysis of HCV RNA. For virus production, transfected cells were seeded into 25-cm^2^ flasks and fed with medium containing 10% FBS. Cells were split every 3–4 days.

### Quantitation of HCV RNA

Total RNA was isolated from cell lysates using an RNeasy kit (Qiagen) in accordance with the manufacturer's instructions. RNA was isolated from cell culture supernatants and gradient fractions (see below) using a QIAamp viral RNA kit (Qiagen). For monitoring RNA replication in transfected cells, we assayed viral RNA abundance in a quantitative real-time RT-PCR reaction carried out in a Bio-Rad iQ5 Real-time PCR Detection System using Taq-Man chemistry and the forward primer HCV84FP (5′-GCCATGGCGTTAGTATGAGTGT-3′), reverse primer, HC300R (5′-CCCTATCAGGCAGTACCACAA-3′), and detection probe: FAM (6-carboxy-fluorescene)-TCTGCGGAACCGGTGAGTACACC-DBH (dual-labeled probe Black Hole Quencher)-1.

### HCV infectivity assays

For virus titration, 100-µl aliquots of serial 10-fold dilutions of supernatant cell culture fluids (clarified by low-speed centrifugation), clarified freeze-thaw cell lysates, or iodixanol or sucrose gradient fractions (see below), were inoculated onto naïve Huh-7.5 cells seeded 24 h previously into 8-well chamber slides (Nalge Nunc) at 3×10^4^ cells/well. Cells were maintained at 37°C in a 5% CO_2_ environment and fed with 200 µl of medium 24 h later. Following 48 h additional incubation, cells were fixed in 1∶1 methanol-acetone at room temperature for 9 min, then stained with monoclonal antibody C7-50 to core protein (Affinity BioReagents, 1∶300) for 2 h at 37°C, washed with PBS twice, and incubated with fluorescein isothiocyanate-conjugated goat anti-mouse immunoglobulin G (Southern Biotech, 1∶100) for 30 min at 37°C. Clusters of infected cells staining for core antigen were considered to constitute a single infectious focus-forming unit (FFU), as described previously [4],[7],[14]. Infectivity titers (FFU/ml) were calculated from the results obtained with sample dilutions yielding 5 to 100 FFU.

### Immunoblotting of viral proteins

Immunoblots of cell lysates were probed with antibody to core (C7-50, Affinity BioReagents; 1∶30,000 dilution), anti-Flag M2 (Sigma, F3165; 1∶1000 dilution), anti-Myc (Sigma, M5546; 1∶1000 dilution) followed by horseradish peroxidase-conjugated anti-mouse IgG (Southern Biotech, no. 1030-05; 1∶30,000). Proteins were visualized by chemiluminescence using reagents provided with the ECL Advance kit (Amersham Biosciences).

### In vitro translation

For in vitro translation of HCV polyprotein segments expressing NS2-NS3-NS4A derived from HJ3 with or without the NS2-S168A mutation, 1 µg of in vitro transcribed RNA was used to program in vitro translation reactions in rabbit reticulocyte lysate (Promega) in a 50 µl reaction mixture containing 2 µl of [^35^S]-methionine (1,000 Ci/mmol at 10 mCi/ml), 2.5 µl of canine pancreatic microsomal membranes and 1 µl of an amino acid mixture lacking methionine at 30°C for the indicated time. Reactions were stopped by the addition of SDS sample buffer and boiling for 10 min. Translation products were separated by SDS-PAGE followed by autoradiography.

### Laser-scanning confocal microscopy

Transfected cells were seeded onto 8-well chamber slides and 2–3 days later washed three times with PBS, fixed with 4% paraformaldehyde, and permeabilized with 0.1% Triton X-100 for 10 min [27]. Cells were labeled with monoclonal antibody C7-50 to core protein (Affinity BioReagents, 1∶400) and rabbit polyclonal antibody to NS5A (a generous gift of Dr. Craig Cameron, 1∶300 dilution) followed by goat anti-mouse immunoglobulin G conjugated to Alexa-488 and goat anti-rabbit immunoglobulin G conjugated to Alexa-594 (Invitrogen, 1∶200). Neutral lipids present in lipid droplets were visualized by staining with LipidTOX Deep Red (Invitrogen). Nuclei were visualized by counterstaining with DAPI (1∶1000). To detect NS2 protein containing Myc- or Flag-tag sequences, cells were incubated monoclonal anti-Myc antibody (Sigma, M5546; 1∶100 dilution) or Rabbit anti-Flag antibody (Sigma, F7425; 1∶100 dilution). E2 protein was detected using the human monoclonal antibody CBH-7 (kindly provided by Steven Foung, Stanford University) at a 1∶100 dilution. Slides were examined with a Zeiss LSM 510 Meta laser-scanning confocal microscope.

### Rate-zonal centrifugation of HCV particles

Cell lysates were prepared for intracellular virus infectivity assays as described by Gastaminza *et al.* [35]. Briefly, cell pellets harvested after trypsinization were resuspended in complete media, washed twice with PBS, lysed by four cycles of freezing and thawing, and clarified by centrifugation at 4000 rpm for 5 mins. A 250 µl volume of lysate was loaded on a preformed continuous 10 to 50% sucrose gradient prepared in TNE (10 mM Tris, pH 8.0, 150 mM NaCl, 2 mM EDTA) and centrifuged for 1 h at 40,000 rpm (∼200,000×g) in a SW41 Ti rotor at 4°C. A total of 40 fractions (300 µl each) were collected from the top of the gradient and subjected to immunoblot analysis, to determine the distribution of core protein, and virus titration to determine the location of infectious virus within the gradient. The density of gradient fractions was estimated from the refractive index determined with a Milton-Roy refractometer.

### Isopycnic gradient centrifugation of HCV particles

Three days following electroporation of cells with HCV RNA, clarified cell lysates (prepared after multiple freeze-thaw cycles as described above) were layered on top of 10 to 40% iodixanol gradients prepared in Hanks balanced salt solution. Gradients were centrifuged in an SW60 rotor (Beckman Coulter) at 45,000 rpm for 16 h at 4°C, and nine fractions (500 µl each) were collected from the top of the tube. The density of gradient fractions was estimated from the refractive index determined with a Milton-Roy refractometer.

### Quantitative core protein ELISA

Core protein was quantified in gradient fractions using the Ortho Trak-C ELISA kit (Ortho-Clinical Diagnostics) with minor modifications to the manufacturer's recommended procedures. Cell culture supernatant or lysates derived from FT3-7 cells transfected with HJ3-5 with or without the NS2-S168G mutation were pre-diluted 10- to 200-fold in the dilution buffer supplied with the kit (so that OD_490_ values were within range of the standard solutions) prior to incubation in the ELISA plates at room temperature for 1 h. The quantity of core protein present in fractions was estimated from the OD_490_ by reference to a standard curve.

### CK2 inhibitor treatment

Cells were treated with the CK2 inhibitor, 2-dimethylamino-4,5,6,7-tetrabromo-1H-benzimidazole (DMAT, EMD Biosciences), as described by Tellinghuisen et al [8]. FT3-7 cells were treated with a range of concentrations (0.1, 1, 10 µM) of DMAT beginning 4 h after electroporation with HCV RNA, with treatment continued until 24 h post-electroporation. We measured the titer of infectious virus present in the cell culture supernatant at 48 h post-transfection.
